# Editorial: Host dynamics and immune evasion: delineating the influence of RNA and DNA viruses

**DOI:** 10.3389/fimmu.2026.1882037

**Published:** 2026-06-23

**Authors:** Won Fen Wong

**Affiliations:** Department of Medical Microbiology, Faculty of Medicine, Universiti Malaya, Kuala Lumpur, Malaysia

**Keywords:** apoptosis, immune remodeling, innate immune sensing, interferon signaling, neuroinflammation, SARS-CoV-2, viral immune evasion

Viruses have evolved alongside their hosts, giving rise to highly sophisticated strategies that promote immune evasion, persistence, and transmission. Central to this evolutionary contest is the ability of viruses to manipulate host immune responses, suppress antiviral defenses, and remodel cellular signaling pathways to establish conditions favourable for survival and replication. Both RNA and DNA viruses deploy diverse mechanisms to interfere with innate sensing pathways, alter immune-cell dynamics, and reshape tissue microenvironments, thereby driving chronic infection, immune dysfunction, and disease progression. Consequently, persistent viral infections are increasingly recognized not merely as infectious disorders, but as complex immunological diseases characterized by sustained remodeling of host immunity and tissue homeostasis.

Despite substantial advances in antiviral therapeutics and vaccination strategies, viral evasion of innate and adaptive immune surveillance remains a major barrier limiting durable antiviral protection and therapeutic efficacy. This challenge is particularly evident in globally significant pathogens, including RNA viruses such as severe acute respiratory syndrome coronavirus 2 (SARS-CoV-2), influenza A virus, hantaviruses, Venezuelan equine encephalitis virus (VEEV), and human immunodeficiency virus type 1 (HIV-1), as well as DNA viruses including human papillomavirus (HPV), all of which continue to contribute substantially to chronic inflammation, immune dysregulation, oncogenesis, and post-viral complications. In this context, the studies assembled in this Research Topic collectively provide important insights into the molecular and cellular mechanisms governing virus–host interactions and viral immune evasion.

A major theme emerging from this Research Topic is the extensive targeting of innate antiviral sensing pathways by viral pathogens. The innate immune system relies on pattern recognition receptors, including Toll-like receptors (TLRs), retinoic acid-inducible gene-I (RIG-I)-like receptors, melanoma differentiation-associated protein 5 (MDA5), and cyclic GMP–AMP synthase-stimulator of interferon genes (cGAS-STING), to detect viral nucleic acids and initiate antiviral responses. However, successful viral pathogens have evolved countermeasures to suppress these pathways. Through the manipulation of host- and viral-derived microRNAs, viruses can disrupt interferon signaling networks and reshape antiviral host defenses, thereby facilitating immune escape (Bayat et al.). Similarly, coordinated inhibition of RIG-I/MDA5-MAVS, TLR3-TRIF, cGAS-STING, and JAK-STAT signaling pathways by HPV E1 proteins profoundly impaired innate antiviral immunity and promoted immune escape in HPV-associated disease, highlighting the remarkable ability of DNA viruses to orchestrate multilayered suppression of host defense systems to establish persistent infection and facilitate tumorigenesis (Li et al.). Furthermore, Betacoronaviral non-structural protein 5 (NSP5)-mediated activation of the cellular sensor DAP5 was shown to drive virus-induced senescence, uncovering a previously underappreciated link between coronavirus infection, host translational control, inflammatory signaling, and cellular aging (Lu et al.).

Several studies within this Research Topic further underscore the importance of chronic immune remodeling during viral infection. HIV-1 persistence remains one of the clearest examples of viral adaptation to host immune pressure. Modulation of interferon regulatory factor (IRF7)-driven transcription was shown to influence HIV-1 latency, emphasizing the central role of interferon regulatory networks in maintaining viral reservoirs (Ezeonwumelu et al.). In parallel, dynamic alterations in circulating γδ T cell populations following antiretroviral therapy during acute HIV-1 infection demonstrated how both viral persistence and therapeutic intervention can reshape immune-cell composition and functionality (Wang et al.). The long-term immunological consequences of viral infection are particularly evident in the context of SARS-CoV-2 and Long COVID. Single-cell immune profiling revealed extensive remodeling of monocytes, natural killer cells, exhausted T cells, and Galectin-9-associated depletion of γδ T cells and mucosal-associated invariant T cells in Long COVID patients with myalgic encephalomyelitis/chronic fatigue syndrome (ME/CFS), providing important mechanistic insights into how persistent immune dysregulation may contribute to chronic post-viral pathology long after apparent viral clearance (Shahbaz et al.). Complementing these observations, retrospective immune profiling demonstrated that hybrid immunity elicited broader neutralizing activity against multiple SARS-CoV-2 variants than vaccination alone, highlighting the need for continual adaptation of vaccination strategies in response to ongoing viral evolution (Mahdi et al.).

Beyond systemic immune responses, several studies highlighted the tissue-specific complexity of antiviral immunity and the intricate cellular interactions that govern disease outcomes. Single-cell RNA sequencing uncovered dynamic and spatially distinct immune-cell populations orchestrating the neuroinflammatory response to VEEV infection within the brain, providing a high-resolution view of the cellular networks that drive viral encephalitis and identifying potential targets for neuroinflammation-directed interventions (Rangel et al.). Extending this concept to systemic viral disease, longitudinal immune profiling of patients with Hantaan virus-induced hemorrhagic fever with renal syndrome revealed phase-specific lymphocyte dynamics and immune signatures predictive of disease severity and progression, emphasizing the importance of temporal changes in cellular immunity during infection (Su et al.). Likewise, divergent macrophage responses during Influenza A virus and *Streptococcus pneumoniae* co-infection revealed that co-infection promoted bacterial dominance through enhanced NF-κB signaling and cell-survival pathways, whereas superinfection favored virus-driven inflammatory priming, highlighting the context-dependent role of macrophage plasticity in shaping disease outcomes (Arranz-Herrero et al.). Collectively, these studies illustrate that antiviral immunity is not dictated by individual immune-cell populations acting in isolation but rather emerges from dynamic interactions among resident and recruited immune cells within distinct tissue microenvironments. In this context, programmed cell death pathways represent another critical interface in virus–host interactions. Influenza A virus was shown to manipulate apoptosis, pyroptosis, necroptosis, and ferroptosis to regulate pulmonary immunity and immunopathology, illustrating how viral pathogens strategically fine-tune host cell fate decisions to optimize replication while limiting complete immune elimination (Luo et al.).

Collectively, the studies featured in this Research Topic underscore the increasingly interdisciplinary nature of contemporary infectious disease research, integrating virology, immunology, genomics, computational biology, and translational medicine. By elucidating the molecular and cellular mechanisms underlying viral immune evasion, chronic inflammation, immune exhaustion, and immunopathology, these contributions substantially deepen our understanding of virus–host interactions. Importantly, these findings provide critical foundations for the development of next-generation antiviral therapeutics, host-directed immunomodulatory strategies, predictive biomarkers, and resilient vaccine platforms capable of confronting both current and future viral threats.

